# Cocultivation Study of *Monascus* spp. and *Aspergillus niger* Inspired From Black-Skin-Red-Koji by a Double-Sided Petri Dish

**DOI:** 10.3389/fmicb.2021.670684

**Published:** 2021-06-09

**Authors:** Xi Yuan, Fusheng Chen

**Affiliations:** ^1^Hubei International Scientific and Technological Cooperation Base of Traditional Fermented Foods, Huazhong Agricultural University, Wuhan, China; ^2^College of Food Science and Technology, Huazhong Agricultural University, Wuhan, China

**Keywords:** *Aspergillus niger*, cocultivation, HPLC, transcriptome, *Monascus* spp., double-sided petri dish

## Abstract

Cocultivation is an emerging and potential way to investigate microbial interaction in the laboratory. Extensive researches has been carried out over the years, but some microorganism cocultivation are not easy to implement in the laboratory, especially the fungus-fungus (FF) cocultivation, owing to the obstacles such as fungal different growth rate, limited growing space, hyphae intertwining, and difficulty of sample separation, etc. In this research, a double-sided petri dish (DSPD) was designed and carried out as a tool to study FF cocultivation in the laboratory. A natural FF cocultivation of *Monascus* spp. and *Aspergillus niger* inspired from black-skin-red-koji (BSRK), were studied. By using DSPD, the aforementioned obstacles in the FF cocultivation study were overcome through co-culturing *Monascus* spp. and *A. niger* on each side of DSPD. The characteristics of monocultured and co-cultured *Monascus* spp. and *A. niger* were compared and analyzed, including colonial and microscopic morphologies, and main secondary metabolites (SMs) of *Monascus* spp. analyzed by high performance liquid chromatography. And a novel SM was found to be produced by *Monascus ruber* M7 when co-cultured with *A. niger* CBS 513.88. Since the above mentioned obstacles, were overcome, we obtained good quality of transcriptome data for further analysis. These results indicate that DSPD might be an efficient tool for investigation of microbial interaction, in particular, for FF interaction.

## Introduction

**FIGURE 1:**
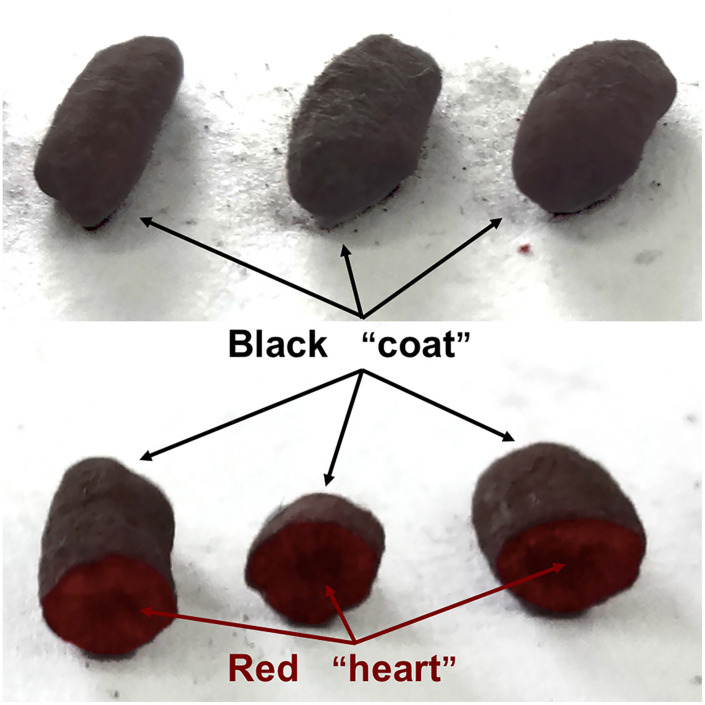
Appearance and cross-sections of black-skin-red-koji (BSRK).

Black-skin-red-koji (BSRK), also known as *Wuyihongqu (乌衣红曲)* in Chinese, is a special traditional Chinese mixed starter which has been utilized for brewing rice wine and cereal vinegar for more than 1,000 years mainly in Fujian and Zhejiang provinces (Fang et al., 2011; Huang et al., 2018). As a representative model of fungus-fungus (FF) cocultivation, the main filamentous fungi in BSRK are *Monascus* spp. and *Aspergillus niger* (Huang et al., 2019). Usually, *A. niger* strains grow on the surface of the steamed rice, and *Monascus* spp. strains grow in the center of the steamed rice, so BSRK has a “red heart” and black “coat” (Figure 1).

*Monascus* spp. belong to the family Monascaceae and were first screened from red yeast rice and characterized by van Tieghem (1884). *Monascus* spp. have been used to produce the food and health products due to their functional secondary metabolites (SMs) such as *Monascus* pigments (MPs), monacolin K (MK), dimerumic acid, and γ-aminobutyric acid (Chen et al., 2015; Feng et al., 2016). However, nephrotoxin citrinin (CIT) can also be produced by a few of *Monascus* spp. strains, which can lead to the food safety concerns of *Monascus* related products (He and Cox, 2016). *A. niger* is most widely used industrially for citric acid production and is also a tool for the production of enzymes such as α-amylase, cellulose, and pectinase (Nielsen et al., 2009; Andersen et al., 2011; Show et al., 2015). However, to date, as a natural FF cocultivation model, the reason why both of *Monascus* spp. and *A. niger* can harmoniously grow in BSRK and the mechanisms relating to how they affect each other are still undiscovered.

Cocultivation is regarded as an experimental imitation of the competition within natural microbe communities in laboratory conditions, microbial cocultivation has been studied for various uses, such as improving SMs production (Wakefield et al., 2017), accelerating the fermentation process (Yan et al., 2008), increasing bioenergy production (Camacho-Zaragoza et al., 2016), speeding up organic pollutants degradation (Kumari and Naraian, 2016), and especially mining novel SMs (Marmann et al., 2014; Yu M. et al., 2019). To date, microbial cocultivation research can be generally classified into three models including bacteria-bacteria (Zhang et al., 2015; Gao et al., 2021), bacteria-fungus (Moussa et al., 2019), and FF (Jimenez-Barrera et al., 2018). Since fungi are one of the biggest sources of natural products and enzymes (Meyer et al., 2016), research on the FF cocultivation has contributed to enhancing SMs production and discovering novel SMs (Shang et al., 2017; Vinale et al., 2017; Yu G. et al., 2019). However, compared to the other models, FF cocultivation is lesser studied, probably due to technical difficulties under laboratory conditions such as fungal different growth rates, limited growth spaces, hyphae intertwining, and difficulty in sample separation, etc.

Genome sequencing technology has revealed that there are a large number of gene clusters regulating SMs synthesis in fungi. However, when fungi are under laboratory culture conditions, most of fungal SMs gene clusters remaines silent (Pettit, 2009). Cocultivation is one of the most efficient methods to activate the silent fungal SMs gene clusters through interspecific communications by co-culture of two or more kinds of fungi in a specific culture environment (Nett et al., 2009; Chiang et al., 2011; Xu et al., 2015).

Since *A. niger* and *Monascus* spp. can be well symbiotic in BSRK, both of them may be an ideal research model of cocultivation. However, usually *A. niger* grows much faster than *Monascus* spp., which has become an obstacle to observing and analyzing the variation between monoculture and co-culture (Favela-Torres et al., 1998). The preliminary results also showed (data not shown) that *Monascus* spp. and *A. niger* could grow together which led to a difficulty in sample separations for further analysis. Furthermore, BSRK and its major fermentation products are all based on rice (Chen et al., 2009; Li et al., 2009), sugars and proteins from rice can cause serious interference to transcriptome sequencing.

**FIGURE 2:**
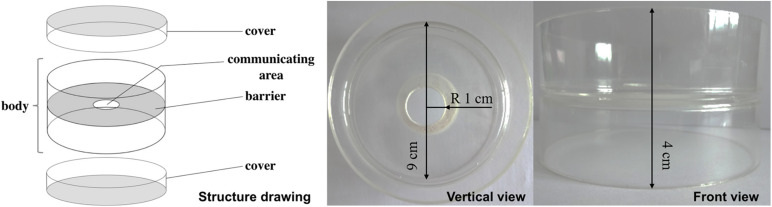
Structure drawing, vertical view, and front view of the double-sided petri dish (DSPD).

In current study, a double-sided petri dish (DSPD, Figure 2) was designed to study the cocultivation of *M. ruber* M7 which can produce high yields of MPs and CIT but no MK, *M. pilosus* MS-1which can produce high yields of MK but no CIT (Feng et al., 2016; Wang et al., 2016), *A. niger* CBS 513.88 which is often used for enzyme production, and *A. niger* CBS 113.46 which is usually applied for citric acid production (Andersen et al., 2011), respectively. The colonial and microscopic morphologies were observed and compared, main SMs were analyzed, novel SMs were mined, and transcriptomes were elucidated, when the aforementioned fungal strains were monocultured and co-cultured on DSPD. The results are proven that DSPD is an efficient tool for FF cocultivation research.

## Materials and Methods

### Strains and Chemicals

The strains *M. ruber* M7 (CCAM 070120, Culture Collection of State Key Laboratory of Agricultural Microbiology, which is part of China Center for Type Culture Collection, Wuhan, China), *M. pilosus* MS-1 (CCTCC M 2013295, China Center for Type Culture Collection, Wuhan, China) and *A. niger* CBS 513.88, *A. niger* ATCC 1015 (Both were purchased from The Westerdijk Fungal Biodiversity Institute) were used in this study. The potato dextrose agar (PDA), potato dextrose broth (PDB), Czapek yeast extract agar (CYA), malt extract agar (MEA), and 25% glycerol nitrate agar (G25N) were taken as culture media. PDB: Potato (200 g/L), sugar (20 g/L), agar power (2 g/L); PDA: PDB with agar power (2 g/L); CYA: NaNO_3_ (3 g/L), K_2_HPO_4_ (1 g/L), KCl (0.5 g/L), MgSO_4_^⋅^7H_2_O (0.5 g/L), FeSO_4_ (0.01 g/L), sucrose (30 g/L), yeast extracts power (1 g/L), agar power (2 g/L); MEA: Malt extract power (30 g/L); G25N: CYA with 25% glycerol. Rice powders agar (RA): rice power (50 g/L), agar power (2 g/L). MK and CIT standard substances were purchased from Sigma Co. Other chemicals were bought from China National Medicines Corporation Ltd.

### Design of a Double-Sided Petri Dish

A double-sided petri dish (DSPD, Figure 2) was produced by two resin petri dishes (ϕ = 9.0 cm). Firstly, the bottoms of two petri dishes were sticked together by glue, then a hole (ϕ = 2.0 cm) in the middle of the petri dishes was drilled. Each DSPD consists of one body part and two covers. When DSPD is used, the metabolites, signal factors, ect. that are produced by the tested microbial strains grown on each side of DSPD can pass and interact through the hole (Xu et al., 2018; Yan et al., 2008). Prior to pouring the media in to DSPD, a round piece of sterile cellophane (ϕ≈ 9.0 cm) is pasted on any side of the DSPD to cover the hole (communicating area) and prevent the media from leaking. After the media solidification on one side, the media are poured on another side.

### Cocultivation Experiments

Two *Monascus* spp. strains were co-cultured with two *A. niger* strains by the following combinations: *M. ruber* M7 and *A. niger* CBS 513.88, *M. ruber* M7 and *A. niger* CBS 113.46, *M. pilosus* MS-1 and. *niger* CBS 513.88, and *M. pilosus* MS-1 and *A. niger* CBS 113.46. All the combinations were investigated by the conventional cocultivation and the cocultivation using DSPD.

### Conventional Cocultivation

**FIGURE 3:**
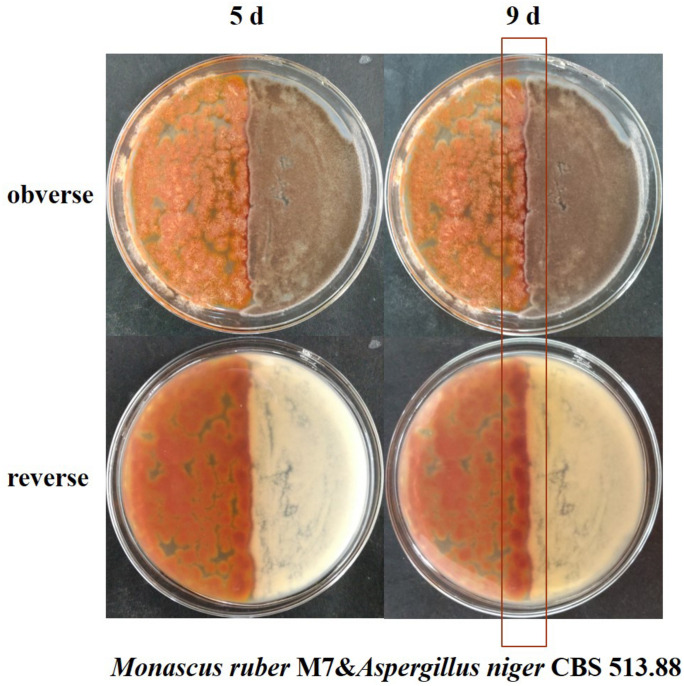
Cocultivation of *Monascus ruber* M7 and *Aspergillus niger* CBS 513.88 by coating inoculation method at 5 and 9 days &-co-cultured with.

**FIGURE 4:**
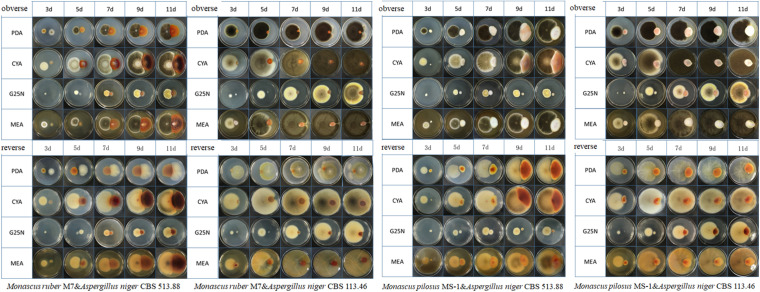
Colonial morphologies of *Monascus ruber* M7, *Monascus pilosus* MS-1 when co-cultured with *Aspergillus niger* CBS 513.88 and *A. niger* CBS 113.46 by conventional (direct) cocultivation at 3, 5,7,9, and 11 days &-co-cultured with.

The conventional cocultivation, means co-culturing two fungal strains in the same petri dish directly (Zuck et al., 2011; Bertrand et al., 2013; Nonaka et al., 2015). Firstly, the strains were cultured on PDA slants at 30 ± 1°C for 7 days and then sterile water was added into the slants and spores were scraped using an inoculating loop. After the mycelia were filtered by sterile filter paper, the spore solution of *Monascus* spp. and *A. niger* were obtained, respectively (Feng et al., 2014). After that, two inoculation methods were used for cocultivation. Coating inoculation: 1 mL *Monascu* spp. and *A. niger* spore solutions were evenly spread on half of the same petri dish, respectively. Point inoculation: 1 μL *Monascus* spp. and *A. niger* spore solutions were inoculated on two points of the same petri dish, respectively, and the distance of the two points was about 2 cm (Shang et al., 2017; Figures 3, 4). After inoculation, all the petri dishes were incubated at 30 ± 1°C for 9 days.

### Cocultivation in DSPD

After the media solidification in two sides, the tested *Monascus* spp. and *A. niger* strains were inoculated on each side of DSPD, respectively. The cocultivation conditions were the same as the conventional cocultivation.

### Observation of Colonial and Microscopic Morphologies

**FIGURE 5:**
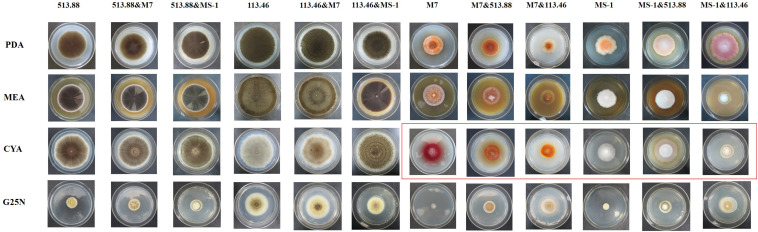
Colonial morphologies of *Monascus ruber* M7, *Monascus pilosus* MS-1 when co-cultured with *Aspergillus niger* CBS 513.88 and *A. niger* CBS 113.46 by using DSPD at 9 days. M7-*M. ruber* M7; MS-1-*M. pilosus* MS-1; 513.88-*A. niger* CBS 513.88; 113.46-*A. niger* CBS 113.46; &-co-cultured with.

**FIGURE 6:**
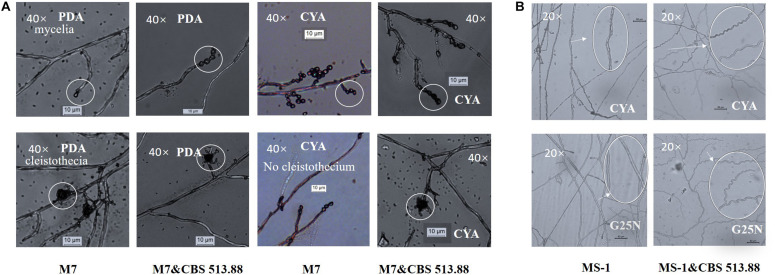
Cocultivation micromorphologies: **(A)** comparison of monocultured and co-cultured (with *Aspergillus niger* CBS 513.88) *M. ruber* M7 on PDA and CYA media; **(B)** mycelia shape variation of *M. pilosus* MS-1 when co-cultured with *A. niger* CBS 513.88. Abbreviations: M7-*M. ruber* M7; M7&CBS 513.88-*M. ruber* M7 co-cultured with *A. niger* CBS 513.88; MS-1-*M. pilosus* MS-1; MS-1&CBS 513.88-*M. pilosus* MS-1 co-cultured with *A. niger* CBS 513.88.

The colonial morphologies of *M. ruber* M7, *M. pilosus* MS-1, *A. niger* CBS 513.88, and *A. niger* CBS 113.46 were observed by the naked eye, and photos were taken by camera at 3, 5, 7, and 9 days (Figures 3–5). The micromorphologies were observed and photos were taken by the microscope (Leica, DM300, Wetzlar, Germany), focusing on mycelia, conidia, and cleistothecia (Figure 6).

### Determination of Color Value of MPs

MPs can be divided into *Monascus* yellow pigment (MYP, 330–450 nm), *M*onascus orange pigment (MOP, 460–480 nm), and *Monascus* red pigment (MRP, 490–530 nm) according to MPs maximum absorption wavelength (Feng et al., 2012; Chen et al., 2017). The color values, which can represent the MPs contents, are defined as optical density values at the given wavelength per milliliter or gram (Feng et al., 2012). Samples were dried by vacuum freeze drying and then weighed (about 1 mg), dissolved by 70% ethanol, and subject to ultrasonic extraction. The extracted solutions were filtrated by filter paper and diluted by 70% ethanol. Diluted solutions were detected by UV–vis UV-1700 Spectrophotometer (Shimadzu, Tokyo, Japan). One unit of optical density at a given wavelength corresponded to one unite (U) of color value (Li et al., 2019):

$$\mathrm{Color}\,\mathrm{value}\,\left(U/g\right)=\frac{A\times \mathrm{dillution}\,\mathrm{ratio}\times V}{m}$$

where *A* is the absorbance of the pigment extract at a given wavelength, *V* is the total volume of extracted solutions, and *m* is the dry weight of the samples used for pigment extraction. The given wavelengths used to detect MYP, MOP, and MRP were 380, 470, and 520 nm, respectively. In addition, 505 nm was taken as the given wavelength to measure total MPs (Ding et al., 2008; Wang et al., 2016).

### Determination of SMs

MK, CIT and MPs varieties were detected by high performance liquid chromatography (HPLC; Feng et al., 2014, 2016; Li et al., 2019). Samples for MK and MPs detection were ultrasonically extracted for 1 h, then centrifugated at 12000 rpm (Heal Force Neofuge 15R, Shanghai, China) for 10 min. Samples for CIT detection were dried and ultrasonically extracted with 80% methanol solvent, then centrifugated at 12000 rpm for 40 min. The supernatants were filtered through a 0.22 μm membrane before HPLC detection. Then MK, CIT and MPs were assessed by HPLC, which were performed on a chromatographic system (Shimadzu, model LC-20A Prominence, Tokyo, Japan), equipped with a diode-array detector (Shimadzu, Tokyo, Japan). The column of inertsil ODS-3 (4.6 mm × 250 mm, id, 5 μm) was employed. For MK detection, the mobile phases were acetonitrile (ACN) and water (contains 0.1% phosphoric acid) (60%:40%, v/v) by isocratic elution, the column temperature was set at 25°C, the flow rate was maintained at 1.0 mL/min and the injection volume was 20 μL. Both acid and lactone forms of MK were calculated as MK yield (Feng et al., 2014). For MPs detection, the mobile phases were ACN and water (contains 0.1% formic acid). Gradient elution was performed as follows: step gradient for ACN was 55% (v/v) to 65% (v/v) in 3 min; 65% (v/v) to 90% (v/v) in 22 min; 90% (v/v) for 5 min; 90% (v/v) to 55% in 1 min; 55% (v/v) for 9 min. The column temperature was kept at 30°C. The detection wavelength was 210 nm to 600 nm. For CIT detection, the mobile phases were ACN and water (contains 0.1% formic acid) (70%:30%, v/v) by isocratic elution, and the flow rate was 1 mL/min. The observation wavelength was 331 nm (Liu et al., 2016). The column temperature was set at 30°C, and the injection volume was 10 μL. When co-cultured with *A. niger*, MK producing strain *M. pilosus* MS-1 was selected to study MK variation while *M. ruber* M7 was selected to study CIT and MPs variation.

### Mining Novel SMs

Our preliminary results (data not shown) found two new SMs from the cocultivation of *M. ruber* M7 and *A. niger* CBS 513.88 in the steamed rice. In this study, DSPD was applied for further research and rice agar was employed to simulated steamed rice. By using DSPD, the mycelia of *M. ruber* M7 were harvested and extracted by ethyl acetate, rotary evaporated, redissolved by 70% ethyl alcohol, then detected by HPLC. The mobile phase condition was ACN and water (contains 0.1% phosphoric acid) (70%:30%, v/v). The observation wavelength was 420 nm according to the maximum absorption wavelength.

### Mycelia Preparation for Transcriptome

DSPD was utilized to separate the tested strains, and cellophane was covered on the surfaces of rice agar. After 5 days of cocultivation, both fungi grew well and the mycelia of co-cultured *M. ruber* M7 and *A. niger* CBS 513.88 were scraped down and collected from the cellophane, respectively. After liquid nitrogen freezing, the mycelia were stored at −80°C and sent to Shanghai Majorbio Bio-pharm Technology Co., Ltd. for transcriptome sequencing.

### RNA Isolation, Library Preparation, and Sequencing

Total RNA was extracted from mycelia using the TRIzol method (Invitrogen, Carlsbad, CA, United States), and the concentration and purity of the extracted RNA were detected by Nanodrop2000. RNA integrity was detected by agarose gel electrophoresis, and the RNA integrity number (RIN) value was determined by Agilent2100. The libraries were constructed after the RNA samples were qualified by using magnetic beads with Oligo(dT) and polyA to pair A-T bases to enrich mRNA. The fragmentation buffer solution was added to randomly break the mRNA into small fragments of about 300 bp. Double-stranded cDNA was synthesized using a SuperScript double-stranded cDNA synthesis kit (Invitrogen, Carlsbad, CA, United States) with random hexamer primers (Illumina). Then the synthesized cDNA was subjected to end-repair, phosphorylation, and “A” base addition according to Illumina’s library construction protocol. The cDNA library was constructed with an Illumina Paired End Sample Prep kit (Illumina, San Diego, CA, United States), quantified by TBS380 (Picogreen, Invitrogen, Carlsbad, CA, United States), and was then sequenced on the Illumina HiSeqTM2500 (2 × 150 bp read length) platform. The transcriptomeic data were analyzed online platform of Majorbio Cloud Platform (www.majorbio.com).

### Statistical Analysis

All experiments were carried out in triplicate. Analysis of variance (ANOVA) was computed for testing the significance of the experiment. Data were analyzed in Microsoft Excel.

## Results

### Comparison of Colonial Morphologies of the Strains on DSPD

Conventional cocultivation of *Monascus* spp. and *A. niger* was carried out at first. Taking *M. ruber* M7 and *A. niger* CBS 513.88 as an example of coating inoculation method (Figure 3), no antagonistic zone can be observed between *M. ruber* M 7 and *A. niger* CBS 513.88. The area color of the *Monascus* spp. colony, which had contacted with *A. niger* CBS 513.88, became redder. By point inoculation method of a different combination of *Monascus* spp. and *A. niger*, there was still no antagonistic zone and the contacted area color was also redder (Figure 4). No significant change in the *A. niger* colony was observed but *A. niger* CBS 113.46 can inhibit the growth of *Monascus* spp. As it was also limited by the growth space, the shape of the colony was irregular. Figure 5 showed that by using DSPD, besides the results observed by conventional cocultivation, all the colonies kept the original shape and the color of the communicating area of *A. niger*, which seemed redder than other areas in the colonies of *Monascus* strains. On the PDA media, the color of co-cultured *M. ruber* M7 was redder than those of the monocultured examples. Especially on CYA media, the colony color of *M. ruber* M7 changed from red to orange when co-cultured with *A. niger* CBS 513.88.

### Comparison of Micromorphologies of the Strains on DSPD

The micromorphologies of monocultured and co-cultured *Monascus* spp. and *A. niger* could hardly compared by conventional cocultivation due to the difficulty in separation and distinguishing of the tested strains. The DSPD solved the problem. The results showed that at the 3rd day, on CYA media, cleistothecia could not be observed in monocultured *M. ruber* M7 but could be observed in co-cultured *M. ruber* M7 (with *A. niger* CBS 513.88) (Figure 6A). At the 7th days, on CYA and G25N media, the mycelia of *M. pilosus* MS-1 changed significantly from straight to curve (Figure 6B). For the micromorphologies of tested *A. niger* strains, no significant change was observed.

### Analysis of SMs From *Monascus* spp. Strains on DSPD

#### Color Value Variation by Cocultivation

**FIGURE 7:**
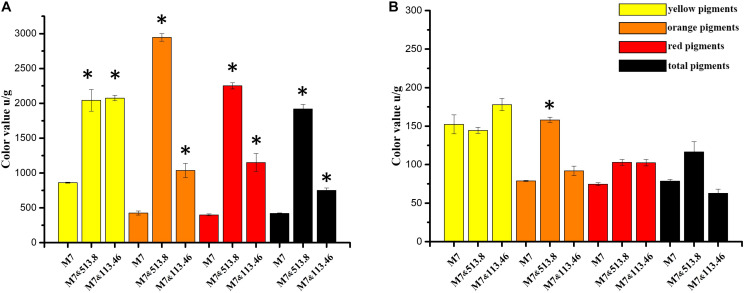
Color value variation of *Monascus* spp. and *Aspergillus niger* cocultivation: **(A)** comparison of *Monascus ruber* M7 intracellular (mycelia) color value among monoculture, co-cultured with *A. niger* CBS 513.88 and co-cultured with *A. niger* CBS 113.46; **(B)** comparison of *M. ruber* M7 extracellular (media) color value among monoculture, co-cultured with *A. niger* CBS 513.88 and co-cultured with *A. niger* CBS 113.46. Abbreviations: M7-monocultured *M. ruber* M7; M7&513.88-*M. ruber* M7 co-cultured with *A. niger* CBS 513.88; M7&113.46-*M. ruber* M7 co-cultured with *A. niger* CBS 113.46. *Represents significant difference compared with control (*P* < 0.05).

As a MP producing strain, *M. ruber* M7 was selected to investigate the changes of color value and MPs (Chen et al., 2017). The results demonstrated a significant change in color value, which reflected the MPs variation of *M. ruber* M7 when co-cultured with *A. niger* strains, respectively. As illustrated in Figure 7, it was clear that both *A. niger* CBS 513.88 and *A. niger* CBS 113.46 could induce *M. ruber* M7 to produce more intracellular (mycelia) MPs (Figure 7A), among which *A. niger* CBS 513.88 acted better than *A. niger* CBS 113.46 in increasing MPs yields. The color value of orange pigments, red pigments, and total pigments increased nearly 6-fold, 4-fold, and 3-fold, respectively. Compared to *A. niger* CBS 513.88, *A. niger* CBS 113.46 was less efficient in the increase of orange and red pigments but was equivalent to an increase of yellow pigments. However, both selected *A. niger* strains had little effect on extracellular (media) MPs of *M. ruber* M7 (Figure 7B).

#### MK and CIT Variation by Cocultivation

MK and CIT are both crucial SMs of *Monascus* spp. As detailed in Table 1, MK was only produced by monocultured *M. pilosus* MS-1 on PDA media. When *M. pilosus* MS-1 was co-cultured with *A. niger* CBS 513.88 and *A. niger* CBS 113.46, respectively MK was undetectable. As a non-CIT producing strain, CIT was undetected in *M. pilosus* MS-1 samples as reported (Feng et al., 2016). According to the CIT content of monocultured and co-cultured *M. ruber* M7, both *A. niger* strains could decrease the intracellular CIT content while increasing the extracellular CIT content of *M. ruber* M7.

**TABLE 1:** Monacolin K and citrinin variation when *Monascus* spp. were co-cultured with *Aspergillus niger* on PDA media.

*Monascus* spp.	Cultivation type	Monacolin K (μg/mg)		Citrinin (μg/mg)	
			
		Intracellular	Extracellular	Intracellular	Extracellular
MS-1	Mono	4.0828	0.4855	Nd	Nd
	Co with CBS 513.88	Nd*	Nd*	Nd	Nd
	Co with CBS 113.46	Nd*	Nd*	Nd	Nd
M7	Mono	Nd	Nd	0.2975	0.0829
	Co with CBS 513.88	Nd	Nd	0.2391*	0.1888*
	Co with CBS 113.46	Nd	Nd	0.2326	0.1231

*Nd: not detected; mono: monocultured; co: co-cultured; ^∗^co-culture group compared to monoculture group, *p*-value ≤ 0.05.*

#### MPs Variation by Cocultivation

**FIGURE 8:**
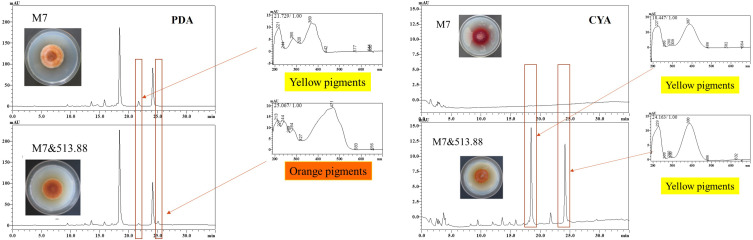
High performance liquid chromatography results of MPs of monocultured *Monascus ruber* M7 and co-cultured *M. ruber* M7 with *Aspergillus niger* CBS 513.88.

As mentioned in the colonial morphologies results (Figure 5), on DSPD we observed that on PDA and CYA media, the colony color of *M. ruber* M7 changed significantly. The HPLC results in Figure 8 could support the above conclusion. The MPs peaks of *M. ruber* M7 can be identified by retention time and UV spectrum according to our previous work (Chen et al., 2017). Under the observing wavelength of 380 nm (Chen et al., 2017), one yellow pigment decreased while one orange pigment increased when *M. ruber* M7 was co-cultured with *A. niger* CBS 513.88 on PDA media. On the CYA media, the colony color changed from red to orange due to the production of more yellow pigments when *M. ruber* M7 was stimulated by *A. niger* CBS 513.88.

### Mining and Primary Localization of Novel SMs by DSPD

**FIGURE 9:**
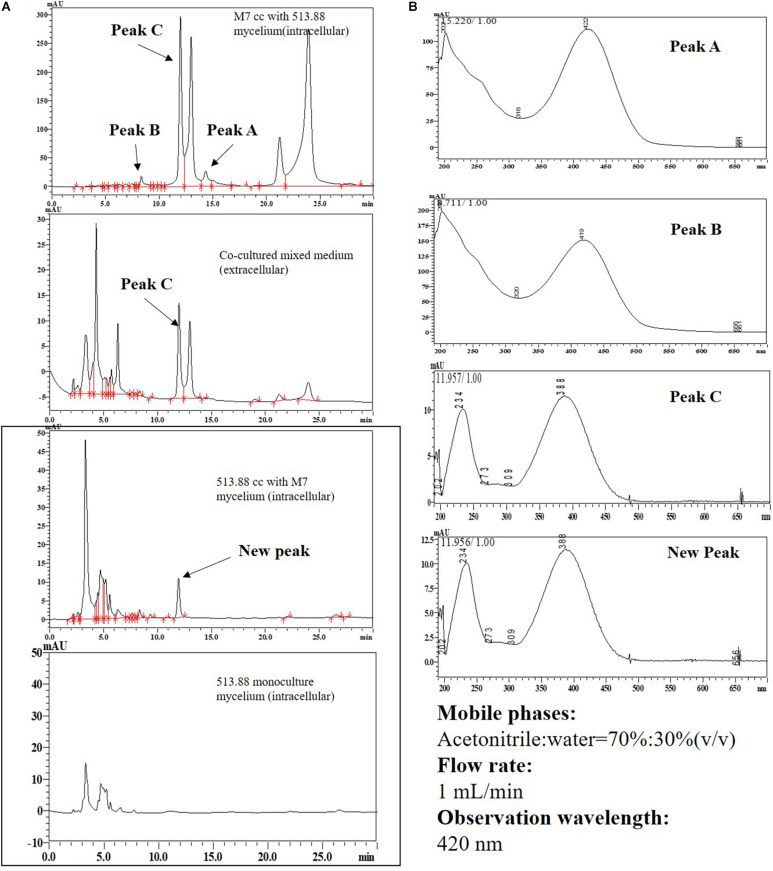
High performance liquid chromatography results of substance A and B in different cocultivation samples: **(A)** HPLC results; **(B)** UV-spectrum results. Abbreviations: CC-cocultured; M7-*Monascus ruber* M7; 513.88- *A. niger* CBS 513.88.

Two novel SMs, namely substance A and B, were discovered by cocultivation of *M. ruber* M7 and *A. niger* CBS 513.88 on the steamed rice in our preliminary experiments (Data not shown). However, the rice-based mixture was not easily analyzed further on account of sample separation. As we saw from Figure 9, when DSPD was applied, substance A can only be detected in the mycelia of co-cultured *M. ruber* M7. Substance B with low yield was detected in culture media and *A. niger* CBS 513.88 mycelia. Compared to monocultured *A. niger* CBS 513.88, a new peak appeared in co-cultured *A. niger* CBS 513.88 mycelia. According to the retention time and UV spectrum, this new peak was similar to peak C that appeared in *M. ruber* M7 mycelia. According to the previous work of our lab (Chen et al., 2017), peak C was identified as a yellow *Monascus* pigment.

### Transcriptomic Analysis of the Strains on DSPD

**FIGURE 10:**
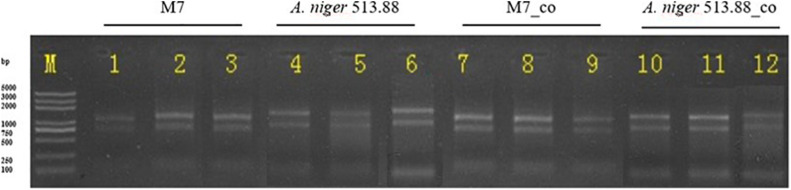
Total RNA of 12 samples for RNA-Seq in 1% agarose gel electrophoresis.

Considering that novel SMs can only be detected in the combination of *M. ruber* M7 and *A. niger* CBS 513.88, and that the genomes of these two fungi have been sequenced or published on NCBI (Andersen et al., 2011). the combination of *M. ruber* M7 and *A. niger* CBS 513.88 was selected for transcriptome analysis. The samples of co-cultured *M. ruber* M7. and co-cultured *A. niger* CBS 513.88 on DSPD were used for total RNA isolation, respectively. Taken samples on the fifth day as an example, in this case monocultured *M. ruber* M7, co-cultured *M. ruber* M7, monocultured *A. niger* CBS 513.88, and co-cultured *A. niger* CBS 513.88, total four samples were chosen as samples to study transcriptome variation during cocultivation. The results of agarose gel electrophoresis (Figure 10) showed that all samples had clear 28S and 18S bands, no significant contamination of proteins, sugars, and other impurities were observed and good RNA integrity was achieved. All samples were in line with sequencing requirements and met the requirements for library construction (Table 2).

**TABLE 2:** The quality results of the total RNA of 12 samples.

Sample	Con (ng/μL)	OD260/280	OD260/230	RIN value
M7_1	112.80	1.96	1.27	9.40
M7_2	180.00	2.05	1.24	9.50
M7_3	245.00	2.05	1.29	10.00
M7_co_1	51.20	2.03	1.12	9.00
M7_co_2	115.60	1.98	1.03	8.20
M7_co_3	130.30	1.90	1.06	9.30
AN5_2	159.60	2.00	1.24	8.60
AN5_3	83.50	2.03	1.73	7.00
AN5_1	73.10	2.05	1.26	8.60
AN5_co_2	285.00	2.02	2.01	8.50
AN5_co_3	301.10	2.02	20.04	8.60
AN5_co_1	715.60	2.25	2.24	6.60

*RIN (RNA integrity number) value represents the integrity of the RNA sample, and the higher the value is, the better the integrity of RNA will be. Abbreviations: Con-Concentration; M7-monocultured *M. ruber* M7; M7_co-*M. ruber* M7 co-cultured with *A. niger* CBS 513.88; AN5-*A. niger* CBS 513.88; AN5_co-*A. niger* CBS 513.88 co-cultured with *M. ruber* M7.*

For *M. ruber* M7 samples, after quality control processing, a total of 46.14 Gb of clean data were obtained. The clean data of each sample reached more than 6.76 Gb. A total of 9,165 genes were detected in this analysis, including 8,671 known genes and 494 new genes. There were 15,985 expressed transcripts, including 8,262 known transcripts and 7,723 new transcripts. The quality values of base Q20 and Q30 were higher than 98% and 95%, respectively, and GC content was higher than 52%, indicating that the data quantity was rich and reliable, which could be used for subsequent analysis in this study. The obtained clean reads were compared with the reported *M. purpureus* genome^1^, and there were six libraries (as listed in Table 3), and more than 95.13% of clean reads in each library could be compared with the reference genome.

**TABLE 3:** RNA-Seq clean reads and the counts, percentage of reads mapping statistics to *Monascus purpureus* genome.

	Sample	Raw reads	Clean reads	Clean bases	Q20 (%)	Q30 (%)	Total mapped	Uniquely mapped
Control	M7_1	48.97 Mb	48.00 Mb	7.17 Gb	98.27	95.03	46.22 Mb (96.31%)	46.04 Mb (95.92%)
	M7_2	58.01 Mb	57.19 Mb	8.54 Gb	98.43	95.36	55.33 Mb (96.75%)	55.10 Mb (96.34%)
	M7_3	51.15 Mb	50.38 Mb	7.55 Gb	98.29	95.00	48.88 Mb (97.02%)	48.67 Mb (96.60%)
Cocultured	M7_co_1	52.72 Mb	51.99 Mb	7.75 Gb	98.47	95.49	49.69 Mb (95.58%)	49.50 Mb (95.22%)
*M. ruber* M7	M7_co_2	57.00 Mb	56.08 Mb	8.37 Gb	98.44	95.42	53.85 Mb (96.02%)	53.65 Mb(95.65%)
transcriptome	M7_co_3	45.76 Mb	44.99 Mb	6.76 Gb	98.41	95.34	42.80 Mb (95.13%)	42.59 Mb (94.66%)

*M7-monocultured *M. ruber* M7; M7_co-*M. ruber* M7 co-cultured with *A. niger* CBS 513.88.*

For *A. niger* CBS 513.88 samples, after quality control processing, a total of 46.33 Gb clean data were obtained, and the clean data of each sample reached more than 7.39 Gb. A total of 12,635 expressed genes were detected in this analysis, including 12,428 known genes and 207 new genes. There were 17,068 expressed transcripts, including 11,653 known transcripts and 5,415 new transcripts. The quality values of base Q20 and Q30 were higher than 98% and 95%, respectively, and GC content was higher than 54%, indicating that the data quantity was rich and reliable, which could be used for subsequent analysis in this study. The obtained clean reads were compared with the reported *A. niger CBS 513.88* genome^2^, and there were six libraries (as listed in Table 4), and more than 93.37% of clean reads in each library that could be compared with the reference genome.

**TABLE 4:** RNA-Seq clean reads and the counts, percentage of reads mapping statistics to *Aspergillus niger* CBS 513.88 genome.

	Sample	Raw reads	Clean reads	Clean bases	Q20 (%)	Q30 (%)	Total mapped	Uniquely mapped
Control	AN5_1	54.46 Mb	53.76 Mb	7.99 Gb	98.30	95.02	50.86 Mb (94.62%)	45.47 Mb (84.59%)
	AN5_2	54.60 Mb	53.90 Mb	8.00 Gb	98.17	94.69	51.22 Mb (95.02%)	45.42 Mb (84.27%)
	AN5_3	51.42 Mb	50.76 Mb	7.52 Gb	98.23	94.87	48.37 Mb (95.29%)	41.94 Mb (82.63%)
Cocultured	AN5_co_1	51.09 Mb	50.46 Mb	7.39 Gb	98.37	95.24	47.12 Mb (93.37%)	42.54 Mb (84.30%)
*A.niger* CBS 513.88	AN5_co_2	52.09 Mb	51.42 Mb	7.67Gb	98.16	94.69	48.79 Mb (94.89%)	46.16 Mb (89.77%)
transcriptome	AN5_co_3	52.74 Mb	52.12 Mb	7.74 Gb	98.30	95.02	49.53 Mb (95.03%)	45.61 Mb (87.51%)

*AN5-*A. niger* CBS 513.88; AN5_co-*A. niger* CBS 513.88 co-cultured with *M. ruber* M7.*

## Discussion

Black-skin-red-koji, as a natural FF cocultivation model, has been applied for wine and vinegar production for over 1,000 years but has been seldom studied (Fang et al., 2011; Mao et al., 2011). In this work, a DSPD was developed and applied to explore BSRK, started from the cocultivation of *Monascus* spp. and *A. niger.* In conventional cultivation of *Monascus* spp. and *A. niger*, we found no antagonism but a symbiosis between *Monascus* spp. and *A. niger* (Figures 3, 4). Moreover, *Monascus* spp. may be stimulated to produce more MPs (Figure 7). Although *Monascus* spp. and *A. niger* can grow together, *A. niger*, especially *A. niger* CBS 113.46 can inhibit the growth of *Monascus* spp. strains. It became more meaningful to lucubrate the reason why *Monascus* spp. and *A. niger* can grow together and has been applied to fermented food for over 1,000 years. Further molecular mechanisms should be studied to reveal the meaning of this cocultivation mode in the biological and food industry. According to the micromorphologic results, at the third day, *A. niger* CBS 513.88 affected *M. ruber* M7 to produce cleistothecia on CYA media. Since cleistothecia are the sexual reproductive organ of *M. ruber* M7 (Ojeda-Lopez et al., 2018), it is speculated that *A. niger* CBS 513.88 might change the proliferation of *M. ruber* M7 under nutrient deficiency conditions. When using PDA as a culture media, there was no significant differences between monocultured *M. ruber* M7 and *M. pilosus* MS-1 on micromorphologic characterics. However, by using CYA and G25N as culture media, *A. niger* can affect the mycelia of *M. pilosus* MS-1 significantly, from straight to curved. The recent research shows that *A. niger* might be stimulated by carbon starving and produce a variety of pectinases which can break cell walls (van Munster et al., 2014). Therefore, considering CYA and G25N are all carbon-starved culture media, it is likely that *A. niger* can be forced to produce pectinase to achieve more carbon resources, meanwhile, the cell wall of *M. pilosus* MS-1 was broken by pectinase and caused a change of mycelia shape (Figure 6B). No significant change was observed by conventional cocultivation of *A. niger*, while on DSPD, the color of the communicating area of *A. niger* was darker than other areas in the colony. Research shows that the black color (melanin) of *A. niger* originated from spores (Kumaran et al., 2017). *Monascus* spp. strains may promote the production of the spores of *A. niger*. The molecular mechanisms about this phenomenon are still being studied.

By conventional cocultivation of fungi, the mycelia are very hard to separate and harvest if there is no antagonism phenomenon between the tested strains, which is an obstacle for SM analysis in this study. DSPD overcame this obstacle efficiently. From MK and CIT results (Table 1), we speculated that *A. niger* may affect the biosynthesis of MK and CIT produced by *Monascus* spp. These results can guide us on transcriptome analysis. With the help of DSPD, more intuitive and completed *Monascus* spp. colony pictures demonstrated the effects of *A. niger* on the MPs production. The above conclusions were further illustrated by the detection of color value and the HPLC analysis of MPs. Both tested *A. niger* can increase the MPs production of *M. ruber* M7, especially the orange pigments. Among them, on increasing MPs of *M. ruber* M7, *A. niger* CBS 513.88 was more efficient (Figure 7). The ultraviolet spectrograms and retention time of MPs peaks from HPLC results were compared to the published research about MPs (Chen et al., 2017), which have demonstrated that *A. niger* can cause variation of several known MPs contents. It was reported that the cocultivation of yeast and *Monascus* spp. and MPs production increased significantly (Shin et al., 1998). This may provide a reference to study the molecular mechanisms about MPs variation. Research has shown that CIT synthesis is related to MPs synthesis (He and Cox, 2016), which may explain the effects of *A. niger* CBS 513.88 on changing the intracellular and extracellular CIT content of *M. ruber* M7.

Research on SMs determination has shown that by using PDA, MEA, CYA, and G25N, no new peak can be observed by HPLC which represents a new compound. This study thus mined novel SMs by other attempts, changing culture temperatures, culture media, and extraction methods. When the rice was taken as a culture media, at 420 nm, two new compounds, namely substance A and B. The chemical structure of A and B are still being identified by LCMS-MS and NMR and will be brought out in the future. DSPD offered an effective method for sample preparation, which is necessary for the preparation and purification of substances A and B. In addition, with the help of DSPD, substance A was almost verified to be produced by *M. ruber* M7 when co-cultured with *A. niger* CBS 513.88. Surprisingly, a peak whose retention time and UV-spectrum were a similar yellow MP, identified by our previous work (Chen et al., 2017), was detected in *A. niger* CBS 513.88 mycelia, indicating that *M. ruber* M7 may stimulate *A. niger* CBS 513.88 to produce MP. These results offer important information for follow-up research. The related experiments on the identification of these new compounds and transcriptome analysis are still ongoing and will be presented in future studies.

To study the most natural co-cultured method of *Monascus* spp. and *A. niger*, which is growing on rice together in a way similar to BSRK, sample preparation represented a huge challenge for transcriptome analyses, due to the separation problems. *Monascus* spp. can grow together with *A. niger* on rice, the mycelia and spores of both sides are intertwined, and difficult to separate from each other. The tested strains were rooted in rice, which made it difficult to separate strains from rice. Rice contains proteins and saccharides which would interfere with the transcriptome sequencing results. The DSPD presented a breakthrough for overcoming this problem. Out of the tested strains, *M. ruber* M7 and *A. niger* CBS 513.88 were chosen as the advisable research subjects for further transcriptome analysis after comprehensive consideration according to the sequenced genome, based on similar growth rate and SMs richness. DSPD broke down the first barrier of transcriptome analysis. All the separation problems about strain-strains and strain-media were solved and good quality transcriptome data were obtained (Tables 4, **5**).

This article reveals some promising superficial results relating to *Monascus* spp. and *A. niger* cocultivation that are worth further exploration. Although we choose *M. ruber* M7 and *A. niger* CBS 513.88 as the main research subjects for some reasonable causes, the results achieved by the DSPD showed that the different pairs of *A. niger* and *Monascus* spp. strains had different interactions, which indicates that it is important to choose appropriate strains for cocultivation to increase beneficial metabolites, decrease detrimental ones, or even find new substances. Beyond the two new substances found by the SMs profiles of *M. ruber* M7 when co-cultured with *A. niger* CBS 513.88, there still may be more undiscovered compounds that were in this case limited by the culture conditions, extraction methods, and instrumental methods. The MPs only changed on yields and not in terms of kind, and the reasons for this on a molecular level are also worth studying in the future. For transcriptome analyses, the present study overcomes the barrier of sample preparation, opening further avenues for the study of the molecular mechanisms of *M. ruber* M7 and *A. niger* CBS 513.88, and obtaining good quality transcriptome results. The DSPD developed in the present study presents an excellent stepping-stone to success. All the above conclusions obtained by DSPD provide the theoretical foundations for further study of the molecular mechanisms between *Monascus* spp. and *A. niger*.

## Data Availability Statement

The datasets presented in this study can be found in online repositories. The names of the repository/repositories and accession number(s) can be found below: NCBI Sequence Read Archive (SRA) with BioProject number PRJNA721011.

## Author Contributions

XY and FC conceived the study and designed the experiments. XY carried out the experiments, analyzed the data, wrote the manuscript, undertook investigation, methodology, writing, reviewing, editing, and supervision. FC reviewed the manuscript and the experiments, oversaw conceptualization, and project administration. Both authors contributed to the article and approved the submitted manuscript.

## Conflict of Interest

The authors declare that the research was conducted in the absence of any commercial or financial relationships that could be construed as a potential conflict of interest.

## Footnotes

1 https://genome.jgi.doe.gov/portal/pages/dynamicOrganismDownload.jsf?organism=Monpu1

2 http://www.aspergillusgenome.org/cache/A_niger_CBS_513_88_genomeSnapshot.html
